# Automatic Segmentation and Quantification of Abdominal Aortic Calcification in Lateral Lumbar Radiographs Based on Deep-Learning-Based Algorithms

**DOI:** 10.3390/bioengineering10101164

**Published:** 2023-10-05

**Authors:** Kexin Wang, Xiaoying Wang, Zuqiang Xi, Jialun Li, Xiaodong Zhang, Rui Wang

**Affiliations:** 1 Department of Radiology, Peking University First Hospital, Beijing 100034, China; 2 School of Basic Medical Sciences, Capital Medical University, Beijing 100069, China; 3 Beijing Smart Tree Medical Technology Co., Ltd., Beijing 102200, China

**Keywords:** abdominal aortic calcification, deep learning, radiography

## Abstract

To investigate the performance of deep-learning-based algorithms for the automatic segmentation and quantification of abdominal aortic calcification (AAC) in lateral lumbar radiographs, we retrospectively collected 1359 consecutive lateral lumbar radiographs. The data were randomly divided into model development and hold-out test datasets. The model development dataset was used to develop U-shaped fully convolutional network (U-Net) models to segment the landmarks of vertebrae T12–L5, the aorta, and anterior and posterior aortic calcifications. The AAC lengths were calculated, resulting in an automatic Kauppila score output. The vertebral levels, AAC scores, and AAC severity were obtained from clinical reports and analyzed by an experienced expert (reference standard) and the model. Compared with the reference standard, the U-Net model demonstrated a good performance in predicting the total AAC score in the hold-out test dataset, with a correlation coefficient of 0.97 (*p* <0.001). The overall accuracy for the AAC severity was 0.77 for the model and 0.74 for the clinical report. Additionally, the Kendall coefficient of concordance of the total AAC score prediction was 0.89 between the model-predicted score and the reference standard, and 0.88 between the structured clinical report and the reference standard. In conclusion, the U-Net-based deep learning approach demonstrated a relatively high model performance in automatically segmenting and quantifying ACC.

## 1. Introduction

Abdominal aortic calcification (AAC) severity is an independent predictor of cardiovascular events and all-cause mortality, particularly in patients with chronic kidney disease [1,2,3]. AAC severity is associated with an increased risk of coronary, cerebrovascular, and cardiovascular disease, and peripheral vascular diseases, regardless of other risk factors [3,4,5,6]. Therefore, the accurate and reproducible quantification of the AAC severity is crucial for diagnosing diseases, determining prognosis, planning treatment, and investigating the effects of drugs [7,8].

The semi-quantification of AAC in lateral lumbar radiographs is useful in assessing AAC severity and is widely applied in clinical practice and many longitudinal studies [9,10]. Aortic wall calcification appears as linear or patchy white areas on radiographs, and can be quantified by radiologists. The Kauppila scoring system is a popular quantification method for evaluating AAC [11]. Since 2015, we have implemented a structured report based on the Kauppila scoring system into our clinical practice, and the results have been used to assess AAC severity, particularly in patients with end-stage kidney disease who have undergone long-term dialysis. However, the identification, localization, and scoring of AAC by radiologists are time-consuming, and the reporting consistency may be only moderate [11], and is exacerbated by the scarcity of trained readers. This prompted us to develop an automatic quantification tool for evaluating AAC. By automatically identifying and quantitatively scoring AAC in clinical practice, we aim to alleviate the workload of radiologists and provide clinicians with objective, reproducible results.

Several studies have attempted to quantify AAC severity using machine learning algorithms [12,13,14,15,16,17]. Deep learning, especially convolutional neural networks, has garnered considerable attention in recent years, particularly regarding image analysis [18,19,20,21,22]. The U-shaped fully convolutional network (U-Net) architecture [23] and its variants have excelled in segmentation tasks [24,25,26,27]. Several studies have demonstrated that deep learning techniques outperform machine learning techniques in automatically scoring aortic calcification [12,28].

In the present study, we aimed to train and test deep-learning-based algorithms to automatically segment and quantify AAC using the Kauppila scoring system in lateral lumbar radiographs. We evaluated our model by comparing the AAC scores generated by the deep learning algorithm with those from routine clinical structured reports and with those assessed by an experienced imaging specialist, which served as the reference standard.

## 2. Materials and Methods

### 2.1. Data Enrollment

**Figure 1:**
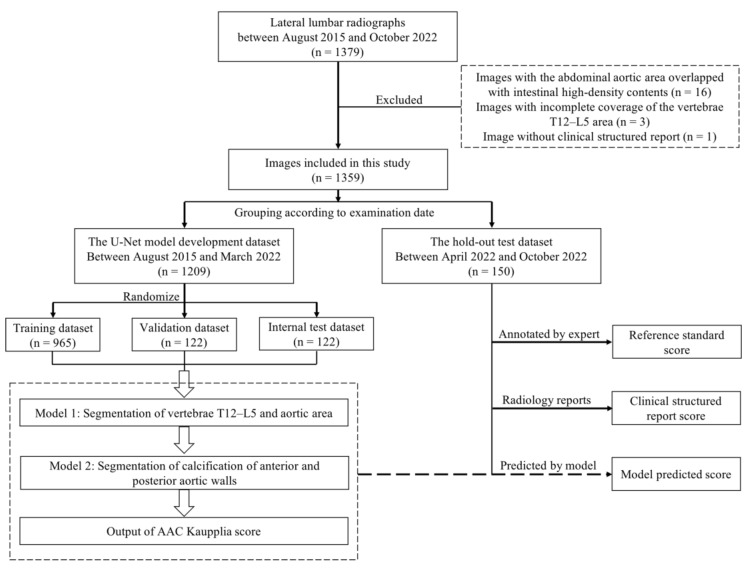
Study flowchart illustrating the data collection and model development. U-Net: U-shaped fully convolutional network; AAC: abdominal aortic calcification.

We retrospectively collected 1379 consecutive lateral lumbar radiographs from our Picture Archiving and Communication System from between August 2015 and October 2022 (Figure 1). After our evaluation, 20 images were excluded based on the following criteria: (a) an abdominal aortic area overlapping with high-density contents in the intestine resulting from ingesting drugs, such as lanthanum carbonate; (b) incomplete coverage of the vertebrae T12–L5 area; and (c) an absence of structured clinical reports. Finally, 1359 lateral lumbar radiographs were included and were divided into two datasets based on the examination date: the model development dataset (comprising radiographs from between August 2015 and March 2022, *n* = 1209) and the hold-out test dataset (comprising radiographs from between April 2022 and October 2022, *n* = 150) The ethics committee of our hospital approved the study protocol (IRB [2019(168)]).

Lateral lumbar radiographs were acquired from patients in the standing position using four radiographic machines from different vendors, as previously described [11]. Table 1 presents the acquisition parameters.

### 2.2. Image Annotation

**Figure 2:**
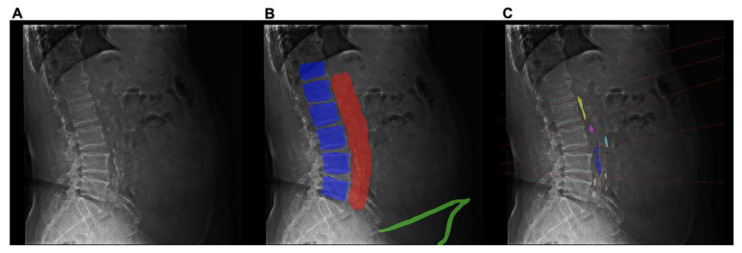
Example of the image annotation and quantification of the abdominal aortic calcification (AAC) Kauppila score in lateral lumbar radiographs. (**A**) The lateral lumbar radiograph of a 56-year-old man diagnosed with stage 5 chronic kidney disease. (**B**) The vertebrae T12–L5 (purple), aortic area (red), calcifications in the anterior (yellow) and posterior (blue) walls of the aorta, and peritoneal dialysis catheter (green) were annotated. (**C**) The AAC was scored following the Kauppila scoring system, with the boundary set as the midpoint of the intervertebral space above and below the vertebrae.

Each lateral lumbar radiograph was annotated pixel by pixel to create image masks for vertebrae T12–L5, the aortic area, and calcifications in the anterior and posterior aortic walls (Figure 2). These annotations were meticulously generated using ITK-SNAP software, specifically version 3.6.0, which can be accessed at www.itksnap.org (accessed on 1 November 2022). The aortic annotation was based on its position relative to the vertebrae. One reader (R1) performed annotations twice, with an interval of at least two months between readings, to obtain a realistic measure of intra-rater reliability for the model development dataset. Subsequently, a radiologist (R2) with 30 years of experience reviewed and revised the annotations. Both the radiologists (R1 and R2) annotated the aortic calcifications and were blinded to the clinical reports. The standard reference scores were calculated from the annotations by the experienced radiologist (R2) and served as a benchmark for evaluating the other scores. The predicted scores were generated using the deep learning model, and the clinical report scores were obtained from structured reports from clinical practice. The model-predicted and clinical report scores were compared with the standard reference scores in the hold-out test dataset.

### 2.3. AAC Scoring

The Kauppila scoring system [11] was used to assess AAC severity. Aortic calcifications in the anterior and posterior walls adjacent to vertebrae L1–4 were scored separately. The boundary was set as the midpoint of the intervertebral space above and below the vertebrae. The AAC was scored as follows: 0 for no AAC; 1 for calcification lengths filling less than 1/3 of the longitudinal aortic wall; 2 for calcification lengths above 1/3 but less than 2/3 of the longitudinal aortic wall; and 3 for calcification lengths above 2/3 of the longitudinal aortic wall. The total AAC scores ranged from 0 to 24. The AAC severity was categorized as mild (total score: 0–4), moderate (total score: 5–15), or severe (total score: 16–24) [29].

### 2.4. Model Development

The X-ray images were anonymized using self-developed C++ software. The patient information in the Digital Imaging and Communications in Medicine file header was replaced with anonymous data following predefined rules. The software processed the Digital Imaging and Communications in Medicine data, applied necessary modifications, and updated the original files to ensure complete anonymization.

The images within the model development dataset were randomly divided into the training (*n* = 965, 80%), validation (*n* = 122, 10%), and internal test (*n* = 122, 10%) datasets. The two-dimensional (2D) U-Net architecture [23] was used to perform AAC segmentation. The 2D U-Net model comprises an encoder–decoder structure with skip connections. The encoder portion extracts features from the input image through a series of down-sampling operations using convolutional layers. In contrast, the decoder up-samples the features back to the original image resolution through a series of up-sampling operations. We downloaded the code of the 2D U-Net model (https://github.com/milesial/Pytorch-UNet/tree/master [accessed on 1 December 2022]) for the image segmentation task. The program was executed on an Nvidia Tesla P100 16G graphics processing unit.

This process was divided into three stages. Initially, a 2D U-Net model was deployed to perform segmentation tasks encompassing the vertebrae (T12–L5) and aortic region within lateral lumbar radiographs. Subsequently, another 2D U-Net model was trained for aortic calcifications, utilizing the aortic label generated in the initial step as the mask. Finally, all labels were used to automatically quantify the Kauppila scores.

In stage one, the images were preprocessed via being resized to 517 × 576 pixels and padded to preserve the original aspect ratio. Portions of the left (40%) and right (10%) sides of each image were cropped. The pixel values were normalized via being scaled linearly with zero-mean normalization within the range from 0 to 1. Augmented images were generated using random scaling (0.95–1.05), image rotation (–10° to +10°), and parallel translation (up to 10% of the image width and height in the horizontal and longitudinal directions). Each of the five epochs was run on 4825 training images in batches of ten. The loss was minimized using the Adam optimizer with a learning rate of 0.0001. The encoder section of the model includes down-sampling convolutional layers with the filter numbers of 64, 128, 256, 512, and 1024. The decoder section has up-sampling convolutional layers with the filter numbers of 512, 256, 128, and 64. The kernel size for all convolutional layers was 3 × 3. These network hyperparameters were empirically determined. The U-Net model was implemented within the PyTorch framework. The Adam optimizer was selected, and the dice similarity coefficient (DSC) was used as the loss function. The loss was continuously monitored during model training. The training process was concluded when a halt in the loss reduction was observed, indicating that a steady state had been achieved.

In stage two, an additional preprocessing step was executed to extract the aortic region from the whole image, based on the previously obtained aortic mask. The output would be a cropped volume that contained only the aortic region of interest as defined by the mask. The model hyperparameters, preprocessing method, and data augmentation were the same as those in stage one.

In stage three, we performed automatic AAC calculations using Python. Firstly, we segmented and located the aortic label in reference to the spine vertebrae. The vertebral labels were divided into six distinct connected domains (T12–L5) and ordered based on their spatial coordinates. Secondly, we determined the vertical direction by calculating the centroids of adjacent connected domains. This information enabled us to create straight lines for segmenting vertebral-connected domains. The initial straight line separated T12/L1, whereas the second line separated L1/2. The aortic label between these lines represented the aorta at the L1 vertebral level. Following this approach, we acquired the aortic region for the levels from L1 to L4. Thirdly, we proceeded to locate calcifications on the aortic wall. We treated the aortic label as a unified entity and established its centerline. Calcifications projecting away from the vertebral direction were classified as anterior wall calcifications, whereas those nearer to the vertebral direction were categorized as posterior wall calcifications. The fourth step involved the computation of AAC scores. After determining the height of each vertebra and aortic wall calcification, we calculated the ratio of the calcification height to the vertebral body height. Subsequently, we applied the Kauppila formula to derive individual vertebral-level AAC scores and the total score. We employed the Python programming language, scientific computing libraries, and image-processing tools for data handling and analysis throughout this computational process. The specific software and packages used included NumPy (https://numpy.org/ [accessed on 1 December 2022]) and SimpleITK (https://simpleitk.readthedocs.io/en/master/ [accessed on 1 December 2022]).

### 2.5. Evaluation of the U-Net Model Performance

The segmentation metrics of the model development dataset were evaluated using the DSC, volume similarity (VS), and Hausdorff distance (HD).

The performance of the automatically predicted AAC scores was evaluated in the hold-out test dataset using the Kendall coefficient of concordance and Pearson’s correlation coefficients pairwise for the structured clinical reports, reference standard, and model-predicted scores. The 95% limits of agreement were used to demonstrate the agreement between the clinical structured reports or model measurements and the reference standards. Additionally, Bland–Altman analysis was conducted to compare the total AAC scores derived from the structured clinical reports and the model-predicted scores with the reference standard AAC scores. Finally, the overall accuracy of the AAC severity, measured via the model and the clinical report, was calculated, using the reference standard as the gold standard.

### 2.6. Statistical Analysis

All statistical analyses were performed using R software (version 4.1.3, R Core Team). Continuous variables are presented as mean ± standard deviation (SD) or the median and interquartile range, depending on their distribution. Categorical variables are presented as percentages. AAC characteristics were evaluated employing the chi-squared (or Fisher’s exact) test for qualitative variables and the Mann–Whitney test for quantitative variables, as appropriate.

The inter-rater reliability and agreement in different score rankings were assessed for ordinal data using the Kendall coefficient of concordance. The total AAC scores were analyzed as a continuous measure and evaluated through pairwise Pearson correlation coefficients to further assess the relationships between the structured clinical reports, reference standards, and model-predicted scores. Additionally, Bland–Altman analysis was performed to examine the agreement and consistency between the structured clinical reports and model-predicted scores compared with the reference standard. Statistical significance was set at *p* < 0.05.

## 3. Results

### 3.1. Clinical Characteristics

Table 2 presents the clinical characteristics and AAC scores of the 1209 cases in the model development dataset. The training, validation, and internal test datasets exhibited a well-balanced distribution of population characteristics, including age and sex (*p* >0.05). The mean age ± SD in the model development dataset was 55.7 ± 13.5 years. Among the study population, 53.4% were men, and 46.6% were women. The AAC severity was fairly well balanced across all datasets (overall: mild, 46.2%; moderate, 31.1%; severe, 22.7%).

### 3.2. Performance of U-Net Models in Model Development

The DSC, VS, and HD values were calculated for the manual annotations and model-predicted labels to quantify the model performance. Table 3 summarizes the performance parameters of the model. The overall DSCs for the vertebrae T12–L5, the aortic area, and calcifications in the anterior and posterior aortic walls were 0.98, 0.98, 0.74, and 0.72, respectively. Correspondingly, the VS values for these labels were 0.99, 0.99, 0.89, and 0.89, respectively, and the HD values were 2.76, 3.64, 23.5, and 20.9 mm, respectively.

A second annotation of the AACs was performed to measure the intra-rater reliability using the hold-out test dataset. The DSC value for the two annotations was 0.79.

### 3.3. Performance of the U-Net Model in AAC Score Quantification

The Kendall coefficients of concordance between the model prediction and the reference standard for the Kauppila scores at vertebrae L1–4 ranged from 0.94 to 0.98. In comparison, the corresponding value between the structured clinical report and the reference standard ranged from 0.81 to 0.90 (Table 4).

**Figure 3:**
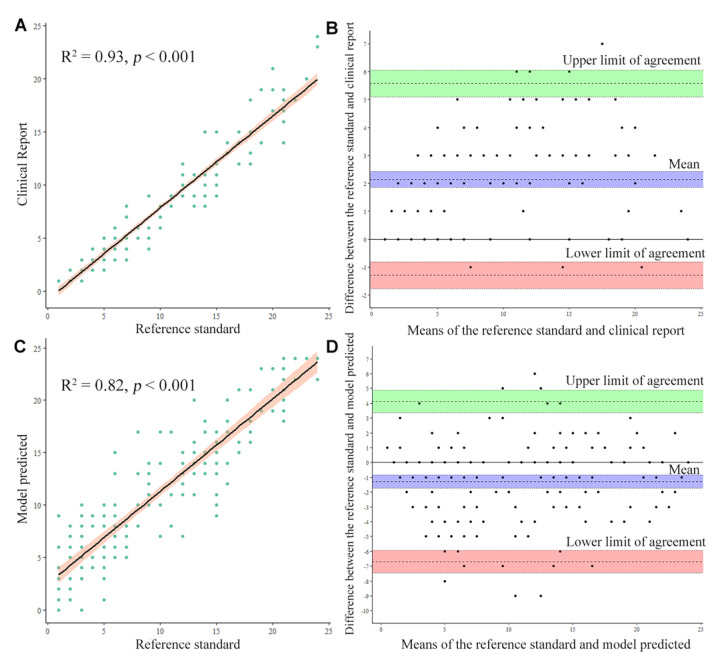
The correlation map and Bland–Altman plots depicting the differences in the total abdominal aortic calcification (AAC) scores between the reference standard and the clinical structured report (**A**,**B**), and between the reference standard and the model-predicted scores (**C**,**D**).

Figure 3 illustrates the U-Net model’s performance in calculating the total AAC score. Pearson’s correlation coefficient between the model prediction and the reference standard for the total AAC score was 0.97, with an adjusted R^2^ of 0.82 (*p* < 0.001). Furthermore, the corresponding value between the structured clinical report and the reference standard was 0.91, with an adjusted R^2^ of 0.93 (*p* < 0.001). The Bland–Altman plots revealed that the average difference between the replicate scores was small and did not increase with the mean AAC score (Figure 3). However, the score variations were smaller in the structured clinical report than in the model prediction. The bias between the structured clinical report and the reference standard was 2.14 (95% confidence interval (CI): from 1.86 to 2.42), and the 95% limits of agreement ranged from −1.77 to 5.57. The bias between the model prediction and the reference standard was −1.3 (95% CI: from –1.75 to –0.85), and the 95% limits of agreement ranged from −7.48 to 4.88.

**Figure 4:**
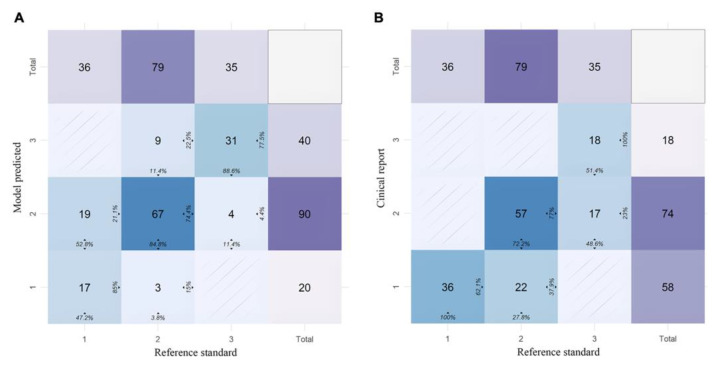
Confusion matrix of the abdominal aortic calcification (AAC) severity derived from the clinical structured report (**A**) and model-predicted scores (**B**), compared with the reference standard.

The accuracy of the AAC severity was 0.77 and 0.74 for the model prediction and structured clinical report, respectively. The Kendall coefficient of concordance for the AAC severity was 0.89 between the model prediction and the reference standard, and 0.88 between the structured clinical report and the reference standard (Table 4). Figure 4 demonstrates the confusion matrix for the clinical structured report and the model prediction of the AAC severity compared with the reference standard.

## 4. Discussion

In our study, we developed a deep-learning-based model to automatically segment and calculate AAC scores using the Kauppila scoring system in lateral lumbar radiographs. Our results demonstrated that the U-Net model can generate automatic and reliable AAC score calculations that are as accurate as the structured clinical reports used in routine practice.

Previous studies have proposed several machine learning techniques. In 2006, Brujne et al. [30,31] employed a shape model with particle filter techniques to automate AAC and evaluated its performance based on accuracy. However, the accuracy of the model can be misleading if the ratio of true negative pixels is unknown. Lauze and Brujne [17] proposed a model combining pixel classification, active shape models, particle filtering, and inpainting for AAC segmentation. However, the best average area overlap with the model was only 0.42. In addition, previous studies did not compare the model-predicted AAC scores with those assigned by radiologists. Petersen et al. [15] reported that appearance models, random forests, and Bayesian models automatically segmented AAC with a correlation coefficient of 0.7 between the model and manual total AAC scores. Our results demonstrated that the U-Net model surpassed the Bayesian model, with a correlation coefficient of 0.97 in the total AAC scores between the model-predicted scores and the radiologist reference standard. Elmasri et al. [16] proposed an appearance model to automatically extract the lumbar vertebrae and aorta based on vertebral fracture assessment images obtained using a dual-energy X-ray absorptiometry scanner. The model was trained on 20 images and tested on another 53 images. Luke et al. [28] used 1600 dual-energy X-ray absorptiometry images to automatically calculate the AAC score, of which only 195 images containing evidence of AAC were included for the model development. They compared the performances of random forest classification and a U-Net model with that of human annotation. Our results showed that the U-Net model outperformed the random forest model, producing reasonable segmentation and AAC scores, but did not perform as well as human annotation. Our study has improved the U-Net model accuracy by using a larger training dataset and producing continuous AAC score measures. This technique yielded an R^2^ coefficient of 0.82, surpassing the 0.49 obtained by Petersen et al. [15] and the 0.59 obtained by Luke et al. [28] for lateral radiographs. Furthermore, another study by Fusaro et al. [32] proposed a semiautomatic tool for quantifying AAC. In their approach, the radiologist used a graphical user interface to draw calcified tracts on radiographs. This enabled the accurate quantification of the calcification length by the software. The results of this study show that the semiautomatic tool can provide scores with a higher reliability and repeatability compared with the subjective scores provided by radiologists. However, one disadvantage of this approach is that radiologists spend approximately 10 min drawing each case, which is time-consuming and limits application in clinical practice. Reid et al. [12] used convolutional neural networks to automatically detect AAC in 1100 vertebral fracture assessment lateral spine images. But among the 1100 images, only 384 images (34.9%) had a total AAC score greater than 5. However, in our study, the AAC severity was fairly well balanced for each dataset and overall (mild [total score: 0–4], 46.2%; moderate [total score: 5–15], 31.1%; severe [total score: 16–24], 22.7%). A recent study by Sharif et al. [14] provided a machine learning model for AAC assessment using 5012 bone-density-machine-derived lateral spine images. The results demonstrated that Pearson’s correlations (r) for different bone density machines between imaging specialist assessments and machine-learning model assessments ranged from 0.78 to 0.91. In our study, Pearson’s correlation coefficient (r) for the total AAC score between the model prediction and the reference standard was 0.97, which is superior to that in the previous study.

Our study has several advantages over previous studies. Firstly, we used a relatively large dataset from different radiographers to train the U-Net model, making the data heterogeneous. This led to better segmentation metrics in our study compared with those in previous studies, with DSCs from 0.72 to 0.74 for calcifications compared with the 0.57 reported in a previous study [28]. Secondly, our predicted scores were comparable to those generated through structured clinical reports, making them suitable for application in clinical settings. Thirdly, previous studies used bone-density-machine-derived lateral spine images, which, incidentally, can be used to measure AAC. This leads to a small sample size of images containing evidence of AAC and a relatively low proportion of images with a moderate and severe AAC severity. In contrast, our study used 1359 standard lateral lumbar radiographs obtained from patients with chronic kidney disease for model development. These are specially used to evaluate AAC severity in patients with chronic kidney disease. Therefore, the majority of our study sample exhibited moderate and severe AAC. Additionally, our method provides the size and density of calcified areas and offers an AAC severity score, enabling the quantification of more subtle differences in the future.

The correlation observed between the automatically generated and manual scores in this study is promising. The comparison of the total AAC score predicted by the model with the reference standard yielded an R^2^ value of 0.82, which is lower than that of the structured clinical report (0.93) but considerably higher than those reported in the literature (0.49–0.68) [28,32]. The discrepancy between the model-predicted score and that of the reference standard may be attributed to the different radiological appearances of AAC, such as discrete plaques with an irregular distribution for intima lesions and linear deposits for the media. The deep learning model should be further trained to improve the accuracy of feature extraction from different calcifications. Additionally, the total AAC scores of the structured clinical report and the reference standard were not similar. This discrepancy may be because radiologists do not always measure the exact lengths of calcifications when assessing images in clinical practice, leading to a reduced accuracy. Furthermore, a single area of calcification bridging the two vertebral sections may not have contributed to either score when assessed manually.

Our study had some limitations. Firstly, this was a single-center retrospective study with a relatively limited sample size, which restricted the generalizability of the results. Secondly, two individuals performed the annotation, which may not be sufficiently robust for a radiographic study, as the annotation of individual pixels containing calcification is subjective. In future, additional data with multiple expert annotations should be included in the segmentation model training. Thirdly, previous studies used dual-energy X-ray absorptiometry images, which are more sensitive to signals from calcification, making it easier for algorithms to extract features from calcification. However, our study used plain radiographs to train and validate our algorithms, which can be easily generalized to plain radiographs. However, they may require tuning for transferal to dual-energy X-ray absorptiometry images, an area that warrants further exploration. Finally, we focused solely on the automatic generation of AAC scores based on the Kauppila scoring system in this study. Future studies should integrate clinical features, laboratory data, and other imaging indicators to generate a more comprehensive prediction system for cardiovascular disease risk scores in patients with chronic kidney disease.

## 5. Conclusions

The use of deep learning algorithms to automatically segment AACs accurately is feasible. The algorithms exhibited an equivalent performance in scoring AACs when compared with experienced radiologists and structured clinical reports. AAC screening can be performed using a fully automated system, eliminating additional imaging or clinician time. The rapid acquisition of AAC scores through automatic quantification may provide new approaches for early identification, disease detection, and cardiovascular event prediction in patients with a high AAC incidence risk in routine clinical practice settings.

## Figures and Tables

**Table 1:** The lateral lumbar radiograph parameters of the model development and hold-out test datasets.

	Training (*n* = 965)	Validation (*n* = 122)	Internal Test (*n* = 122)	Hold-Out Test (*n* = 150)	Overall (*n* = 1359)
Vendor					
Carestream health, n (%)	322 (33.4%)	41 (33.6%)	38 (31.1%)	126 (84.0%)	527 (38.8%)
GE healthcare, n (%)	383 (39.7%)	53 (43.4%)	47 (38.5%)	23 (15.3%)	506 (37.2%)
Kodak, n (%)	190 (19.7%)	19 (15.6%)	25 (20.5%)	0 (0%)	234 (17.2%)
Siemens, n (%)	70 (7.3%)	9 (7.4%)	12 (9.8%)	1 (0.7%)	92 (6.8%)
FOV, mm^2^	424 [424, 424]	424 [404, 424]	424 [404, 424]	410 [404, 424]	424 [404, 424]
Tube current, mA	250 [250, 250]	500 [250, 789]	499 [250, 766]	499 [250, 510]	498 [250, 630]
Tube voltage, kV	40.0 [26.0, 58.0]	32.0 [19.0, 45.5]	35.0 [22.0, 52.0]	35.5 [22.0, 55.3]	35.0 [22.0, 54.0]
Pixel spacing, mm	0.14 [0.14, 0.14]	0.14 [0.14, 0.19]	0.14 [0.14, 0.19]	0.15 [0.14, 0.19]	0.14 [0.14, 0.19]

FOV: field of view.

**Table 2:** The clinical characteristics and abdominal aortic calcification (AAC) scores of the model development dataset.

	Overall (*n* = 1209)	Training (*n* = 965)	Validation (*n* = 122)	Internal Test (*n* = 122)	*p* Value
Sex					0.94
Men, n (%)	646 (53.4%)	514 (53.3%)	67 (54.9%)	65 (53.3%)	
Women, n (%)	563 (46.6%)	451 (46.7%)	55 (45.1%)	57 (46.7%)	
Age, years	55.7(13.5)	55.7 (13.3)	54.8 (13.0)	56.1 (15.3)	0.86
Peritoneal dialysis catheter					0.41
Yes, n (%)	355 (29.4%)	286 (29.6%)	30 (24.6%)	39 (32.0%)	
No, n (%)	854 (70.6%)	679 (70.4%)	92 (75.4%)	83 (68.0%)	
AAC score					
L1 anterior score	0 [0, 1]	0 [0, 1]	0 [0, 1]	0 [0, 0]	0.47
L1 posterior score	0 [0, 1]	0 [0, 1]	0 [0, 1]	0 [0, 1]	0.56
L2 anterior score	0 [0, 2]	0 [0, 2]	0 [0, 2]	0 [0, 2]	0.75
L2 posterior score	0 [0, 2]	0 [0, 2]	0 [0, 2]	0 [0, 2]	0.86
L3 anterior score	1 [0, 3]	1 [0, 3]	1 [0, 3]	0 [0, 3]	0.32
L3 posterior score	0 [0, 2]	0 [0, 2]	0 [0, 2]	0 [0, 2]	0.25
L4 anterior score	0 [0, 3]	1 [0, 3]	1 [0, 3]	0 [0, 3]	0.22
L4 posterior score	1 [0, 3]	1 [0, 3]	1 [0, 3]	0 [0, 2]	0.06
Total AAC score	6 [0, 15]	6 [0, 15]	8 [0, 15]	3 [0, 14]	0.20
AAC severity					0.33
Mild: 0–4, n (%)	559 (46.2%)	439 (45.5%)	53 (43.4%)	67 (54.9%)	
Moderate: 5–15, n (%)	376 (31.1%)	302 (31.3%)	42 (34.4%)	32 (26.2%)	
Severe: 16–24, n (%)	274 (22.7%)	224 (23.2%)	27 (22.1%)	23 (18.9%)	

**Table 3:** The evaluation metrics for the segmentation models in the model development dataset.

	Overall	Training	Validation	Internal Test
DSC				
Vertebrae T12–L5	0.98 [0.97, 0.98]	0.98 [0.98, 0.99]	0.96 [0.92, 0.97]	0.95 [0.91, 0.97]
Aorta	0.98 [0.96, 0.98]	0.98 [0.97, 0.98]	0.94 [0.90, 0.95]	0.93 [0.89, 0.95]
Posterior wall calcification	0.74 [0.58, 0.82]	0.77 [0.66, 0.84]	0.44 [0.14, 0.60]	0.56 [0.03, 0.65]
Anterior wall calcification	0.72 [0.59, 0.80]	0.75 [0.66, 0.81]	0.52 [0.29, 0.64]	0.53 [0.18, 0.65]
VS				
Vertebrae T12–L5	0.99 [0.99, 1.00]	0.99 [0.99, 1.00]	0.98 [0.95, 0.99]	0.98 [0.94, 0.99]
Aorta	0.99 [0.98, 1.00]	0.99 [0.99, 1.00]	0.97 [0.95, 0.99]	0.97 [0.94, 0.99]
Posterior wall calcification	0.89 [0.79, 0.95]	0.91 [0.82, 0.95]	0.81 [0.52, 0.92]	0.74 [0.52, 0.92]
Anterior wall calcification	0.89 [0.81, 0.95]	0.90 [0.82, 0.95]	0.84 [0.66, 0.94]	0.82 [0.65, 0.91]
HD (mm)				
Vertebrae T12–L5	2.76 [2.04, 6.97]	2.48 [2.00, 3.35]	15.7 [5.40, 36.2]	22.2 [6.23, 34.4]
Aorta	3.64 [2.79, 8.21]	3.31 [2.68, 4.08]	16.1 [8.95, 35.4]	17.4 [8.96, 36.7]
Posterior wall calcification	23.5 [9.20, 49.0]	19.6 [6.76, 38.6]	48.2 [26.2, 72.8]	35.7 [16.3, 74.2]
Anterior wall calcification	20.9 [8.96, 45.8]	17.5 [7.49, 42.9]	35.7 [22.5, 51.9]	33.3 [17.1, 54.8]

DSC: Dice similarity coefficient, VS: volume similarity, HD: Hausdorff distance.

**Table 4:** Evaluation of the model’s performance in abdominal aortic calcification (AAC) score quantification in the hold-out test dataset.

	Reference Score	Structured Clinical Report		Model Prediction	
		Score	Correlation Coefficient	Score	Correlation Coefficient
L1 anterior wall			0.98		0.81
0	80 (53.3%)	82 (54.7%)		88 (58.7%)	
1	28 (18.7%)	42 (28.0%)		20 (13.3%)	
2	32 (21.3%)	20 (13.3%)		21 (14.0%)	
3	10 (6.7%)	6 (4.0%)		21 (14.0%)	
L1 posterior wall			0.97		0.83
0	74 (49.3%)	75 (50.0%)		81 (54.0%)	
1	29 (19.3%)	41 (27.3%)		17 (11.3%)	
2	26 (17.3%)	20 (13.3%)		27 (18.0%)	
3	21 (14.0%)	14 (9.3%)		25 (16.7%)	
L2 anterior wall			0.96		0.82
0	58 (38.7%)	61 (40.7%)		40 (26.7%)	
1	32 (21.3%)	50 (33.3%)		28 (18.7%)	
2	36 (24.0%)	24 (16.0%)		37 (24.7%)	
3	24 (16.0%)	15 (10.0%)		45 (30.0%)	
L2 posterior wall			0.97		0.84
0	65 (43.3%)	70 (46.7%)		60 (40.0%)	
1	26 (17.3%)	40 (26.7%)		24 (16.0%)	
2	37 (24.7%)	25 (16.7%)		27 (18.0%)	
3	22 (14.7%)	15 (10.0%)		39 (26.0%)	
L3 anterior wall			0.96		0.85
0	46 (30.7%)	54 (36.0%)		43 (28.7%)	
1	35 (23.3%)	49 (32.7%)		20 (13.3%)	
2	27 (18.0%)	17 (11.3%)		25 (16.7%)	
3	42 (28.0%)	30 (20.0%)		62 (41.3%)	
L3 posterior wall			0.96		0.84
0	50 (33.3%)	56 (37.3%)		51 (34.0%)	
1	30 (20.0%)	52 (34.7%)		20 (13.3%)	
2	40 (26.7%)	25 (16.7%)		37 (24.7%)	
3	30 (20.0%)	17 (11.3%)		42 (28.0%)	
L4 anterior wall			0.94		0.87
0	49 (32.7%)	60 (40.0%)		34 (22.7%)	
1	25 (16.7%)	50 (33.3%)		21 (14.0%)	
2	33 (22.0%)	15 (10.0%)		29 (19.3%)	
3	43 (28.7%)	25 (16.7%)		66 (44.0%)	
L4 posterior wall			0.95		0.9
0	37 (24.7%)	43 (28.7%)		50 (33.3%)	
1	21 (14.0%)	49 (32.7%)		19 (12.7%)	
2	40 (26.7%)	26 (17.3%)		23 (15.3%)	
3	52 (34.7%)	32 (21.3%)		58 (38.7%)	
AAC severity			0.89		0.88
1	36 (24.0%)	58 (38.7%)		20 (13.3%)	
2	79 (52.7%)	74 (49.3%)		90 (60.0%)	
3	35 (23.3%)	18 (12.0%)		40 (26.7%)	
Total AAC score	9 [5, 15]	6 [3, 12]	0.97	10 [6, 16]	0.91

## Data Availability

The data are available upon request.

## References

[1] Tatami Y., Yasuda Y., Suzuki S., Ishii H., Sawai A., Shibata Y., Ota T., Shibata K., Niwa M., Morimoto R. (2015). Impact of abdominal aortic calcification on long-term cardiovascular outcomes in patients with chronic kidney disease. Atherosclerosis.

[2] Kidney Disease: Improving Global Outcomes (KDIGO) CKD-MBD Update Work Group (2017). KDIGO 2017 Clinical practice guideline update for the diagnosis, evaluation, prevention, and treatment of chronic kidney disease-mineral and bone disorder (CKD-MBD). Kidney Int. Suppl..

[3] Leow K., Szulc P., Schousboe J.T., Kiel D.P., Teixeira-Pinto A., Shaikh H., Sawang M., Sim M., Bondonno N., Hodgson J.M. (2021). Prognostic value of abdominal aortic calcification: A systematic review and meta-analysis of observational studies. J. Am. Heart Assoc..

[4] Wong N.D., Lopez V.A., Allison M., Detrano R.C., Blumenthal R.S., Folsom A.R., Ouyang P., Criqui M.H. (2011). Abdominal aortic calcium and multi-site atherosclerosis: The multiethnic study of atherosclerosis. Atherosclerosis.

[5] Zhang H., Li G., Yu X., Yang J., Jiang A., Cheng H., Fu J., Liang X., Liu J., Lou J. (2023). Progression of vascular calcification and clinical outcomes in patients receiving maintenance dialysis. JAMA Netw. Open.

[6] Schousboe J.T., Taylor B.C., Kiel D.P., Ensrud K.E., Wilson K.E., McCloskey E.V. (2008). Abdominal aortic calcification detected on lateral spine images from a bone densitometer predicts incident myocardial infarction or stroke in older women. J. Bone Miner. Res..

[7] Cox A.J., Hsu F.C., Agarwal S., Freedman B.I., Herrington D.M., Carr J.J., Bowden D.W. (2014). Prediction of mortality using a multi-bed vascular calcification score in the Diabetes Heart Study. Cardiovasc. Diabetol..

[8] Lewis J.R., Schousboe J.T., Lim W.H., Wong G., Wilson K.E., Zhu K., Thompson P.L., Kiel D.P., Prince R.L. (2018). Long-term atherosclerotic vascular disease risk and prognosis in elderly women with abdominal aortic calcification on lateral spine images captured during bone density testing: A prospective study. J. Bone Miner. Res..

[9] Wilson P.W., Kauppila L.I., O’Donnell C.J., Kiel D.P., Hannan M., Polak J.M., Cupples L.A. (2001). Abdominal aortic calcific deposits are an important predictor of vascular morbidity and mortality. Circulation.

[10] Niu Q., Zhao H., Wu B., Tsai S., Wu J., Zhang M., Lu L., Qiao J., Men C., Zuo L. (2019). Abdominal aortic calcification is superior to other arteries calcification in predicting the mortality in peritoneal dialysis patients—A 8 years cohort study. BMC Nephrol..

[11] Kauppila L.I., Polak J.F., Cupples L.A., Hannan M.T., Kiel D.P., Wilson P.W. (1997). New indices to classify location, severity and progression of calcific lesions in the abdominal aorta: A 25-year follow-up study. Atherosclerosis.

[12] Reid S., Schousboe J.T., Kimelman D., Monchka B.A., Jafari Jozani M., Leslie W.D. (2021). Machine learning for automated abdominal aortic calcification scoring of DXA vertebral fracture assessment images: A pilot study. Bone.

[13] Conrad-Hansen L.A., de Bruijne M., Lauze F., Tankó L.B., Pettersen P.C., He Q., Chen J., Christiansen C., Nielsen M. (2007). Quantifying calcification in the lumbar aorta on X-ray images. Med. Image Comput. Comput. Assist. Interv..

[14] Sharif N., Gilani S.Z., Suter D., Reid S., Szulc P., Kimelman D., Monchka B.A., Jozani M.J., Hodgson J.M., Sim M. (2023). Machine learning for abdominal aortic calcification assessment from bone density machine-derived lateral spine images. EBioMedicine.

[15] Petersen K., Ganz M., Mysling P., Nielsen M., Lillemark L., Crimi A., Brandt S.S. (2012). A Bayesian framework for automated cardiovascular risk scoring on standard lumbar radiographs. IEEE Trans. Med. Imaging.

[16] Elmasri K., Hicks Y., Yang X., Sun X., Pettit R., Evans W. (2016). Automatic Detection and Quantification of Abdominal Aortic Calcification in Dual Energy X-ray Absorptiometry. Procedia Comput. Sci..

[17] Lauze F., de Bruijne M. (2007). Toward automated detection and segmentation of aortic calcifications from radiographs. Proc. SPIE.

[18] Gao Z., Pan X., Shao J., Jiang X., Su Z., Jin K., Ye J. (2022). Automatic interpretation and clinical evaluation for fundus fluorescein angiography images of diabetic retinopathy patients by deep learning. Br. J. Ophthalmol..

[19] Fu F., Shan Y., Yang G., Zheng C., Zhang M., Rong D., Wang X., Lu J. (2023). Deep learning for head and neck CT angiography: Stenosis and plaque classification. Radiology.

[20] Ao J., Shao X., Liu Z., Liu Q., Xia J., Shi Y., Qi L., Pan J., Ji M. (2023). Stimulated Raman Scattering Microscopy Enables Gleason Scoring of Prostate Core Needle Biopsy by a Convolutional Neural Network. Cancer Res..

[21] Lu S., Ding Y., Liu M., Yin Z., Yin L., Zheng W. (2023). Multiscale Feature Extraction and Fusion of Image and Text in VQA. Int. J. Comput. Intell. Syst..

[22] Li M., Ling R., Yu L., Yang W., Chen Z., Wu D., Zhang J. (2023). Deep learning segmentation and reconstruction for CT of chronic total coronary occlusion. Radiology.

[23] Ronneberger O., Fischer P., Brox T. (2015). U-Net: Convolutional networks for biomedical image segmentation. Med. Image Comput. Comput. Assist. Interv..

[24] Zair A.M., Bouzouad Cherfa A., Cherfa Y., Belkhamsa N. (2023). An automated segmentation of coronary artery calcification using deep learning in specific region limitation. Med. Biol. Eng. Comput..

[25] Rajamani K.T., Rani P., Siebert H., Elagiri Ramalingam R., Heinrich M.P. (2023). Attention-augmented U-Net (AA-U-Net) for semantic segmentation. Signal Image Video Process..

[26] Sun Q., Dai M., Lan Z., Cai F., Wei L., Yang C., Chen R. (2022). UCR-Net: U-shaped context residual network for medical image segmentation. Comput. Biol. Med..

[27] Zhang H., Zhong X., Li G., Liu W., Liu J., Ji D., Li X., Wu J. (2023). BCU-Net: Bridging ConvNeXt and U-Net for medical image segmentation. Comput. Biol. Med..

[28] Luke C., Tim C. (2019). Automated scoring of aortic calcification in vertebral fracture assessment images. Proc. SPIE.

[29] Verbeke F., van Biesen W., Honkanen E., Wikström B., Jensen P.B., Krzesinski J.M., Rasmussen M., Vanholder R., Rensma P.L., CORD Study Investigators (2011). Prognostic value of aortic stiffness and calcification for cardiovascular events and mortality in dialysis patients: Outcome of the calcification outcome in renal disease (CORD) study. Clin. J. Am. Soc. Nephrol..

[30] Bruijne M.D. Shape particle guided tissue classification. Proceedings of the 2006 Conference on Computer Vision and Pattern Recognition Workshop (CVPRW’06).

[31] Bruijne M.D. (2005). A pattern classification approach to aorta calcium scoring in radiographs. Computer Vision for Biomedical Image Applications—CVBIA 2005.

[32] Fusaro M., Schileo E., Crimi G., Aghi A., Bazzocchi A., Barbanti Brodano G., Girolami M., Sella S., Politi C., Ferrari S. (2022). A novel quantitative computer-assisted score can improve repeatability in the estimate of vascular calcifications at the abdominal aorta. Nutrients.

